# Farming Animals in Extreme Poverty: A Problem for Anti-speciesists?

**DOI:** 10.1007/s11406-025-00834-9

**Published:** 2025-04-14

**Authors:** Joshua Jarvis-Campbell

**Affiliations:** Department of Social and Political Sciences, Philosophy, and Anthropology, University of Exeter, Exeter, UK

**Keywords:** Extreme poverty, Animal agriculture, Demandingness, Lifeboat ethics, Speciesism, Animal ethics

## Abstract

Some philosophers argue that animals shouldn’t be given less moral consideration simply on the basis of their species membership. These “anti-speciesists” argue that many common practices involving animals are morally objectionable, animal agriculture being one of their most common targets. However, it is questionable whether the same objections apply to those who farm animals in extreme poverty. Anti-speciesists tend to accept such a practice, arguing that it is permissible because it may be necessary for meeting the basic needs of those who engage in it. I argue that, despite the argument’s intuitiveness, it may be problematic for those who think humans and animals deserve similar levels of moral consideration. In particular, it forces the anti-speciesist into a trilemma: they can either accept the permissibility of farming animals in extreme poverty while also conceding that it would be permissible to farm and kill humans in certain circumstances, reject the permissibility of farming animals in extreme poverty, or abandon the anti-speciesist position. I argue that the first two options are not as problematic for the anti-speciesist as they might first appear. For this reason, I argue, farming animals in extreme poverty does not present a (major) problem for anti-speciesists.

## Introduction

While racism involves discriminating against others on the basis of race, and sexism involves discriminating against others on the basis of sex, speciesism involves discriminating against others on the basis of species membership. Like racism and sexism, some philosophers have argued that speciesism is a morally untenable position (Regan, 2004; Singer, 2015). We cannot, they argue, give preferential treatment to the interests of humans over nonhuman animals simply in virtue of being human. Yet most practices involving animals today do exactly that. We experiment on them to test cosmetic products, we hunt them for fur, and we keep them in barren enclosures for our entertainment. Furthermore, we subject billions of animals to confined, unsanitary living conditions in order to consume them later (CIWF, n.d.). The suffering inflicted on animals in industrial animal agriculture is enormous. Yet we continue to farm animals in this way, even though such a practice is not necessary for our health or gustatory pleasure.

“Anti-speciesists” hold that we cannot do this. Animals, they argue, deserve serious moral consideration in our moral decision making, and their interests cannot be discounted simply in virtue of their species membership. Such anti-speciesist sentiments can be found in a wide variety of normative positions, including utilitarianism (Singer, 2015), rights-based ethics (Regan, 2004), virtue ethics (Hursthouse, 2011), contractarianism (Rowlands, 1997), and care ethics (Donovan & Adams, 1996). Despite the wide range of views held by anti-speciesists, I believe the majority of them will be susceptible to the problem I present in this paper.

The problem stems, I argue, from anti-speciesist responses to other forms of animal agriculture. Consider the large number of people living in extreme poverty^1^ who engage in animal agriculture.^2^ For these people, abstaining from animal agriculture may not be such an easy task. Intuitively, it seems like these people do not do wrong when they engage in such a practice. If the anti-speciesist position implies that these people are doing something morally objectionable, then this could be a problem for the anti-speciesist position.^3^

However, anti-speciesists tend to grant the moral permissibility of farming animals in extreme poverty. They argue that it can be justified because such a practice is necessary for meeting the basic needs of those who engage in it:^4^

In extreme cases of survival, the killing and eating of animals must be considered permissible… it is completely unreasonable to expect people to sacrifice themselves in order to respect another’s interests. (Cochrane, 2012, p. 57)

If it were true that these nutrients [for human health and vitality] were not otherwise obtainable, then the case for eating meat, even given the rights view, would be on solid ground. (Regan, 2004, p. 337)

When there is an irreconcilable conflict between the basic survival needs of animals and of normal humans,^5^ it is not speciesist to give priority to the lives of [normal humans]. (Singer, 2011, p. 122).

Despite the intuitive plausibility of such an argument, I will argue that it is problematic for the anti-speciesist. It is problematic, so I argue, because it entails that farming and killing humans in certain circumstances would also be morally permissible. I argue that the anti-speciesist who defends farming animals out of necessity cannot avoid this problem, and so they face a trilemma: they can either accept the permissibility of farming animals in extreme poverty while also conceding that it would be permissible to farm and kill humans in certain circumstances, reject the permissibility of farming animals in extreme poverty, or abandon the anti-speciesist position.

I will argue that the first and second options are potential ways out of this trilemma for the anti-speciesist. I will do this by briefly defending two controversial positions: 1) it is plausible that human farming can be justified in certain circumstances, and 2) it is plausible that farming animals in extreme poverty is not morally permissible. These arguments are not intended to demonstrate the truth of either option 1 or 2, but they can serve to show that both options are not as unpalatable as they might first appear. I conclude, for this reason, that farming animals in extreme poverty does not present a major problem for the anti-speciesist.

## Anti-speciesism and the Argument From Necessity

Consider the situation of people living in extreme poverty. According to the United Nations, such a condition can be “characterized by severe deprivation of basic human needs, including food, safe drinking water, sanitation facilities, health, shelter, education and information” (UN, 1996, p. 38). Animal agriculture, for many of these people, provides a means of meeting some of these fundamental needs. Some animal products can reduce stunting and certain micronutrient deficiencies, while others can promote skeletal, muscular, and neurological development (Banda & Tanganyika, 2021, p. 9). Livestock can also provide a source of income, allowing farmers to purchase other foods or essential goods. Other benefits of livestock can include a source of draught power, manure for fertilizer, and a buffer against risk (Upton, 2004, p. iv).

Without animal agriculture, many of these people may not be able to meet their financial or nutritional needs. To abstain from such a practice thus risks financial insecurity, malnutrition, or even death. To say that one ought to abstain from such a practice, even if it results in malnutrition or death, will strike many as being absurd. As such, the anti-speciesist argues, those who must farm and kill animals are justified in doing so. It is permissible because it is necessary for meeting the basic needs of those who engage in it.

It is not my intention to fully assess the plausibility of this argument. Instead, my focus will be on one problematic implication that follows from this argument. The problem, which many anti-speciesists appear not to have considered,^6^ is that if farming and killing animals can be justified in cases of necessity, then the same reasoning could be used to justify farming and killing humans in cases of necessity. There have been many instances throughout history whereby people have engaged in homicidal cannibalism out of necessity. One famous example is that of R v Dudley and Stephens, a case concerning 4 sailors adrift at sea on a small lifeboat (Hutchinson, 2010, pp. 13–40). After many days without food or water, two sailors, Dudley and Stephens, killed Parker, the cabin boy, and consumed his blood and flesh. They did this because they believed it was necessary for them to survive. The case concluded, however, that necessity did not justify (let alone excuse) their actions. A precedent was set: against the charge of murder, necessity is not a defence.

While there is plenty of evidence of homicidal cannibalism throughout history, evidence of farming humans seems slim. Nevertheless, it is not difficult to imagine some dystopian future where humans are regularly farmed for their organs, and where some people have to engage in such a practice in order to meet their basic needs. If necessity is sufficient to justify farming and killing animals, even if such animals deserve serious moral consideration, then why couldn’t the same apply to humans?

The anti-speciesist could respond by arguing that there are significant differences between farming animals in cases of necessity and farming humans in cases of necessity. For instance, an anti-speciesist could argue that humans would experience far more suffering as a result of such a practice due to their greater intellectual abilities, or that there would be wider negative effects on society if such a practice were to occur. An interest-based rights anti-speciesist on the other hand may argue that humans have an intrinsic interest in freedom that animals lack (Cochrane, 2012, pp. 49–52), and as such are unique in having a right not to be confined on a farm. Others may argue that there is something significantly more repulsive about farming humans than farming animals, much in the same way people find the idea of consuming human flesh to be far more repulsive than consuming animal flesh.^7^ If such differences are morally relevant, then they could serve to explain why farming and killing animals in cases of necessity would be permissible, while farming and killing humans in cases of necessity would be impermissible.

The problem with these suggestions however is that these differences between farming humans and farming animals are contingent. If farming humans out of necessity is impermissible just for these reasons, then such a practice would be permissible if these reasons were controlled for. This is, in a sense, trivially true, but it has important implications. For instance, consider the claim that farming and killing humans, unlike farming and killing animals, would be impermissible, even if necessary to meet one’s basic needs, because humans would experience far more suffering in such a practice. One reason for this might be because humans, due to their greater intellectual capacities, would have the ability to comprehend the awful situation they are in and feel despair. But what if we constructed a hypothetical where this difference is eliminated? Suppose the humans being farmed are distracted with enough delicious food and video games that they would experience just as much pleasure and pain as a non-factory farmed animal today. Would it then be permissible to kill them and sell their organs, provided it was the only way for me to feed and clothe myself for a year? Clearly, many will say, such a practice is wrong. But, if the anti-speciesist thinks it would be permissible to farm and kill animals out of necessity, and the supposed moral difference between farming humans and farming animals has been controlled for (greater suffering in this case), then the anti-speciesist would be forced to concede that farming and killing humans would also be permissible if the amount of suffering was equivalent.

Another example will help illuminate the problem. Consider the claim that farming and killing humans, unlike farming and killing animals, would be impermissible, even if necessary to meet one’s basic needs, because it would cause far greater negative side effects. One reason for this is that many people will be saddened and scared to think that such a horrible practice occurs in the world. In response, we can construct a hypothetical where humans of a particular skin colour are farmed as a result of extreme racism. Suppose that those who are not farmed are not saddened by such a practice because they share extremely racist views. Furthermore, they are not worried about being potentially farmed themselves because the structure of their society makes this impossible to happen. In such a world, the negative side effects (to the non-farmed group) will have been reduced to an extent that mirrors the negative side effects of farming animals. Yet such a practice would still be wrong, even if these negative side effects were controlled for. But the anti-speciesist will have to concede that such a practice would be permissible if a similar practice of farming and killing animals out of necessity is permissible.

I hope these examples serve to show the problem the anti-speciesist faces. If they wish to claim that it would be wrong to farm humans in cases of necessity, but not animals, then they have to put forward some kind of reason for thinking this is the case. For any reason they put forward however (e.g. humans would experience greater suffering), a hypothetical can be put forward in which that difference does not obtain. If the anti-speciesist wishes to rely on this reason for thinking that farming and killing humans out of necessity is impermissible while farming and killing animals out of necessity is permissible, then they will need to concede that it would be permissible to farm and kill humans out of necessity if this reason is controlled for. In other words, they would have to grant that it would be permissible to essentially engage in human slavery under certain conditions. This seems to be a major problem for the anti-speciesist.

## Some Objections

Several objections could be raised against my argument at this stage. Firstly, it could be argued that there are some significant moral differences between farming animals in cases of necessity and farming humans in cases of necessity that cannot be controlled for. For instance, one could argue that only the latter practice involves farming beings which belong to the same species as us. Perhaps we have some special obligations to beings which belong to the same species as us, much in the same way as we have special obligations to those who are closest to us (e.g. family, friends, etc.).

It is true that this feature cannot be controlled for. But it is difficult to see how an anti-speciesist can put forward this position. One could use the same reasoning to say that we have special obligations to members of our own race. But this seems to be the position of a racist, not an anti-racist. Similarly, to say one has special obligations to members of one’s own species seems to follow primarily from a speciesist, as opposed to anti-speciesist, position.

Another objection is that my examples of farming humans is incoherent because it is inefficient.^8^ Presumably, the humans being farmed would need to eat the same food as the farmers. But in that case, the food for the farmed humans can be eaten directly by the farmers. As such, there would be no need to farm humans, because a farmer’s nutritional needs could be met by eating the human “feed” directly. Animals, on the other hand, can survive off plants that are inedible to humans, and as such it may be necessary to farm them in order to meet the farmer’s nutritional needs.

This objection makes a mistake in assuming that the cases of necessity under discussion only cover nutritional needs. Anti-speciesists who accept the permissibility of killing animals for nutritional needs would also likely accept killing animals for other important needs. For instance, they would hold that it is permissible for a farmer to farm and sell the flesh of animals if this were the only way for her to afford medicine which she needs to survive. But now suppose that the farmer can only afford the medicine needed to survive by farming humans and selling their organs. Unlike a situation where humans are farmed for nutrition, farming humans in this example is not inefficient, and so is coherent. The objection above, therefore, does not get anti-speciesists out of the problem. So long as they maintain that farming animals out of necessity is permissible, they cannot deny the permissibility of farming humans in ways that are coherently necessary for survival.

A final objection one could raise involves arguing that the problem above is not even a problem for the anti-speciesist because it only involves conceding that it would be permissible to farm and kill humans in an absurd hypothetical. Clearly, the above hypotheticals will never occur in the real world. In which case, to dismiss the anti-speciesist position in light of what it would entail in some imaginary world seems absurd.

It is true that the anti-speciesist is only forced to concede the permissibility of farming and killing humans in an absurd hypothetical, but this nevertheless means that they have to admit that human slavery is justified in certain circumstances. If this absurd hypothetical were to obtain, the anti-speciesist would have no argument against farming humans in such circumstances. A position which holds that slavery can be justified in certain circumstances seems weaker than a position which completely condemns the practice, and so the anti-speciesist position appears weaker for this reason. As such, this is a problem that cannot be so easily dismissed.

## A Trilemma

The anti-speciesist has three options here. The first option is to maintain that farming animals in cases of necessity is permissible and concede that farming humans in certain circumstances would also be permissible. The second option is to deny the permissibility of farming humans in cases of necessity and thus accept that farming animals in cases of necessity would also be impermissible. The third option is to abandon the anti-speciesist position and argue that there is a moral difference between the two practices because humans and their interests deserve greater moral consideration simply in virtue of being human. Clearly, none of these options will be appealing to the anti-speciesist, and so they face a trilemma.

I will argue that, despite initial appearances, option 1 and option 2 are both plausible options for the anti-speciesist. I cannot make a full case in favour of either option here, but I can at least demonstrate that they are not as unpalatable as they might first appear.

### Why Human Farming in Certain Cases May Be Morally Permissible

If option 1 is to be defended, I will need to argue that it is at least plausible to suggest that farming humans in some cases of extreme poverty may in fact be permissible. Despite the *prima facie* difficulty of such a task, I believe it can be done by properly considering what such a practice would actually entail. A hypothetical which controls for all the potential reasons as to why farming humans out of necessity is worse than farming animals out of necessity would be so radically different that we may simply be unable to comprehend how such a practice may not be so bad after all. That is, it is difficult to even imagine what farming humans out of necessity would look like if we removed all of the factors that make it worse than farming animals out of necessity. If a comprehensive picture of such a practice were made, perhaps one would think that the practice is in fact permissible. In what follows, I try to paint such a picture.

Consider, first, the reasons an anti-speciesist might give for thinking that farming humans is worse than farming animals. They might argue that humans would suffer more as a result of their greater intellectual abilities, that farming humans would produce worse societal consequences, or that such a practice would involve violating certain rights of humans that animals may lack. What would farming humans look like if these reasons were properly controlled for? Here is one such example:

Suppose that, around the world, humans have always been farmed. Not just any humans though. The humans being farmed are children, “bred” thousands of years ago for the purpose of being farmed for their organs. These “farm children” are quite content moving about the farm (which is more like a playground), playing with other farm children, and eating meals provided by the farmers. They seem completely uninterested in the outside world. Unlike other humans, their cognitive capacities suddenly stop developing at the age of 3, and they have a natural lifespan of just 20 years. They are painlessly killed, however, just before the age of 3. After which, their organs are sold. Other humans do not seem disturbed by the practice. There is no fear of other humans being farmed because the line between farm children and other humans has been clearly demarcated.

In such a hypothetical, all of the suggested reasons the anti-speciesist may give for thinking that farming humans is worse than farming animals have been controlled for. Nevertheless, it seems likely that many philosophers would reject such a practice, hereafter referred to simply as “children farming”. However, the anti-speciesist does not need to argue in favour of children farming *generally*. They merely need to argue that such a practice can be plausibly justified *in cases of necessity*. Suppose there are some people who need to engage in children farming in order to meet their basic needs. Can their actions be plausibly justified?

I will present two arguments the anti-speciesist could use to defend children farming in cases of necessity. My intention is not to provide a full defence of either argument. Instead, I merely want to suggest that the anti-speciesist *could* employ one (or both) of these arguments to try to show that such a practice can be plausibly justified. The first argument appeals to the beneficial consequences of such a practice. The second argument appeals to the overdemandingness of abandoning such a practice.

Consider, first, the consequences of engaging in children farming in cases of necessity. Such a practice is of course good for the farmers, but one could also argue that it is good for the children as well. This is because they wouldn’t have existed if it weren’t for the practice. That is, even if the practice involves killing them at a young age, it nevertheless gives them an existence they wouldn’t otherwise have. Better to exist and be killed than never exist at all, one could argue.

One might argue in response that it seems impossible to compare existence to non-existence, and as such we cannot coherently say that existence is “better” for the children than non-existence.^9^ However, we don’t need to argue that existence is better for these children to get this argument off the ground. It seems plausible to say that it is “bad” for a being to be brought into existence if that being experiences nothing but agony throughout their lives. Similarly, it seems plausible to say that it is “good” for a being to be brought into existence if they have a life worth living (McMahan, 2008, p. 68; Singer, 2015, p. 228). “Good” and “bad” are, in such cases, being used in a non-comparative sense. In which case, it seems plausible to say that, so long as the children have lives worth living, the practice of children farming is on-the-whole good for the children because it gives them a life they would not otherwise have.

The anti-speciesist can then argue that since the practice is good for the children, and since it allows farmers to meet their basic needs and thus avoid great suffering, then it should be permitted.^10^ After all, consider the two choices the farmer faces:

(a) Bring farm children into existence and kill them later, thus giving life to these children and preventing great suffering for the farmer

(b) Don’t bring farm children into existence, thus giving no life to these children and the farmer experiences great suffering

Between these two choices, the consequences of (a) appear far better^11^ than (b). On these grounds, the anti-speciesist could argue that children farming in cases of necessity can be plausibly justified. This is not to say that the argument is without problems,^12^ but it does provide at least some ground for thinking that justifying human farming in cases of necessity is not as implausible as it initially appeared.

Another way anti-speciesists could try to justify children farming in cases of necessity is by appealing to demandingness. If farmers need to farm these children in order to meet their basic needs, then it would be extremely demanding for these farmers to give up such a practice. If they didn’t engage in children farming, they would risk financial insecurity, malnutrition, or even death. Perhaps asking them to give up so much would be asking more than what morality can reasonably demand. That is, morality may be limited in what it can demand, and if so, then it may be permissible for these farmers to engage in children farming out of necessity.

The idea that morality is limited in what it can demand is intuitively plausible. If morality is extremely demanding, then this may entail that we have an obligation to give away all of our possessions, or sacrifice our own lives, if doing so saved others. But this seems absurd. Morality, it seems, is simply not that demanding. In which case, perhaps children farming can be justified on the grounds that prohibiting such a practice would be unreasonably demanding.

One may object however that appealing to demandingness does not work in the case of children farming. Concerns around demandingness are usually raised as a response to demanding *positive* duties, that is, duties of assistance. We typically believe that we don’t have extremely demanding positive obligations, e.g. an obligation to sacrifice ourselves for the benefit of others. Refraining from children farming, on the other hand, is a *negative* duty – a duty we have to refrain from harming others. It is common to think that these negative duties are far more stringent.

Suppose, for instance, that a famine has struck, and George and I find ourselves on the verge of starving to death. Let’s suppose that I have some food left – just enough to keep me alive until help arrives. George, on the other hand, has no food. Do I have an obligation to give George my food, even if this results in my death? While it may be morally praiseworthy to give George my food, it does not seem like I have a moral obligation to do this. An explanation for why this may be the case is that it would be extremely demanding for me to give up my food. Demandingness seems to limit our positive obligations to one another.

Suppose now that the situation is reversed, so that only George has enough food to survive. Would it be permissible to kill George, given that this is the only way for me to survive? The answer, it seems, is obviously no. I have very strong negative duties to refrain from harming George, even if fulfilling these negative duties is extremely demanding. The same may be said about farming children. Even if morality is limited in what it can demand, such limitations may only apply to our positive, as opposed to negative, duties.

The first thing to point out in response to this argument is that there are many instances in which we *do* think one’s negative duties change depending on how demanding it would be to abide by them (Lichtenberg, 2010, pp. 572–575). We typically think it is permissible for people to drive cars if they have no other means of transportation, even if doing so increases the risk to other people of getting run over or of being harmed by pollution. As such, the idea that overdemandingness only limits our positive duties is questionable.

Of course, this does not show that it is permissible to kill other humans if not doing so would cost us our lives. This is because there is obviously a large difference between a weak duty not to do something which risks killing someone, such as driving, and a strong duty not to intentionally kill another human. But if we are willing to grant that weak negative duties can be overridden if they are too demanding to follow, then it should be at least plausible to think that strong negative duties can also be overridden provided the sacrifice required of us is high enough.

Is the sacrifice required of a children farmer high enough to justify killing a farm child? The example above involving George seems to suggest it isn’t. Even if I have a right to life, this does not entail that I have a right to harm or use another’s body for my survival (Thomson, 1971). But now suppose that George only has a week left to live before dying painlessly (and that I would die of starvation before this point). If I were to kill him, then I would (only) be depriving him of a week of life. Because of this, one could argue that killing George does not cause too much harm. This could be done by appealing to a *deprivation account* of the harm of death, which holds that death is bad insofar as it deprives us of good things in the future (Nagel, 1970). Now, it seems clear that I have, in general, a strong negative duty not to kill people who will die a week later anyway. But given how demanding it would be for me not to kill George, one could argue that I am under no obligation to follow this strict duty given how much suffering I would experience were I to follow it.

One might object at this stage that this does nothing to show that it is plausible to kill farm children out of necessity, because the farm children are deprived of far more life than George when killed. But it could be argued that even if they are deprived of many years of life, they are not harmed as much by death as an “ordinary” human. This can be seen when we consider arguments put forward (some of which by anti-speciesists) as to why death is generally worse for humans than it is for animals.

Tom Regan, for instance, argues that the harm caused by death “increases as the number and variety of possible sources of satisfaction increases” (Regan, 2004, p. xxxiv). Humans, he argues, can experience a greater variety of sources of satisfaction than animals because of their greater psychological capacities.^13^ For instance, Regan suggests that only humans can derive satisfaction from being moral agents—they can receive satisfaction from thinking impartially about moral choices (Regan, 2004, pp. xxxiv-xxxv). Other examples of satisfaction exclusive to humans may include the satisfaction one gets from reading, creating music, and having deep conversations with others. Death, on Regan’s account, may be significantly worse for most humans than it is for animals because death would foreclose a greater variety of sources of satisfaction for humans. If the lower psychological capacities of animals mean that they have few sources of satisfaction, then perhaps death is not so bad for them when compared to the death of a human. Similarly, if farm children have lower psychological capacities (compared to an average adult human), then their limited sources of satisfaction may mean that their death is not so bad for them on Regan’s account.

Another argument as to why death may be worse for humans than animals (and thus farm children) employs what we may call a *desire-thwarting account* of the badness of death. According to this account, death is bad for a being because it frustrates plans and goals they have regarding the future (Li, 2002, pp. 33–42). Some philosophers argue that animals lack the psychological ability to contemplate or care about the far future (McMahan, 2008, p. 67). For this reason, they have no long-term desires or ambitions for the future that would be frustrated by death. In which case, death for animals may not be so bad. Similarly, since farm children are killed before the age of 3, their mental life may be more similar to animals who live moment to moment, not caring about the far future. In which case, death for these children may not be so harmful according to the desire-thwarting account.

As such, even if a farm child is deprived of many years of life by being killed at a young age, their death may not be so significant when compared to the death of a farmer. If death is not so bad for farm children, and if it would be extremely demanding for farmers to give up such a practice, then the anti-speciesist could argue that it would be permissible to kill farm children in cases of necessity.

The anti-speciesist, then, could try to argue that children farming in cases of necessity can be plausibly justified by employing one (or both) of these arguments. Again, it is important to emphasize that I am not providing a full defence of either argument. Both arguments are likely susceptible to a variety of objections. However, if either of the above arguments are at least *plausible*, then we should at least conclude that the permissibility of farming humans in certain cases of necessity is not as implausible as it first appeared.

What does this mean for the anti-speciesist? Recall that if the anti-speciesist thinks it is permissible to farm and kill animals out of necessity, but not permissible to farm and kill humans out of necessity, then they need to provide a reason to explain this difference without appealing to species membership. However, for any reason put forward, a hypothetical scenario could be created which controls for this difference. The problem for the anti-speciesist is that they would be forced to concede that in such a hypothetical, it would be permissible to farm and kill humans. However, the preceding discussion has shown that upon close consideration of what such a hypothetical would entail, it may not be so implausible to make such a concession. That is, one could admit that in the absurd hypothetical, it may in fact be permissible to farm and kill humans. In which case, the anti-speciesist could maintain their position in thinking that farming and killing animals in extreme poverty is morally permissible, while conceding that it would be permissible to farm and kill humans in very bizarre circumstances. But since the concession has been shown to be at least not implausible, the force of the problem is significantly lessened.

### Why Farming Animals in Cases of Necessity May Be Impermissible

The above argument entails that it is at least plausible to think that farming humans in certain cases of necessity is morally permissible. However, it is very likely that not every anti-speciesist will agree. Farming and killing humans, they might say, is still impermissible even in such an absurd situation. Controlling for many reasons against the practice, showing how it may be good for the humans, and emphasizing the demandingness of abstaining from it, makes little difference to the moral permissibility of the practice. However, an anti-speciesist which held this position would have no ground for also asserting the moral permissibility of farming animals in cases of necessity. In which case, they would have to deny the permissibility of both practices (option 2), or abandon the anti-speciesist position (option 3).

I believe that a strong case can be made for thinking that option 2, despite initial appearances, is a plausible option for the anti-speciesist. Taking this option involves conceding that it would be impermissible to farm and kill animals in cases of necessity. There are some anti-speciesists who have defended such a position. Gary Francione, for instance, has argued that it is always wrong to harm an innocent being, even in cases of necessity (Francione, 2021). This may be the case if animals have extremely stringent rights that should rarely (if ever) be infringed.^14^

Whether or not animals do have such stringent rights cannot be decided here. But what can be noted is that many philosophers have argued that *humans* have such rights. That is, they have argued that humans have rights that are so stringent it would be impermissible to infringe on them, even if doing so is necessary for saving one’s own life (Frowe, 2014, p. 46; Otsuka, 2003, p. 68; Thomson, 1991, p. 290).^15^ For instance, Michael Otsuka asks us to imagine a javelin is headed towards me. The only way for me to save myself is to grab an innocent stranger and use their body as a shield. Otsuka claims that this use of a bystander is *clearly* not permitted, even if it is necessary for saving my life. If it is the case that humans have rights that are so stringent that it would be impermissible to kill them even to save oneself, then the anti-speciesist could make a strong case for thinking that the same rights apply to animals. This strikes me as a plausible option.

One may nevertheless find this position problematic given its troubling implication that farming animals in extreme poverty would be morally impermissible. To say that these farmers are acting wrongly, even if they need to farm animals in order to meet their basic needs, is undoubtedly an uncomfortable thought. But it should be emphasised that if these farmers are doing something morally problematic, that doesn’t mean they should be blamed or punished for their actions. Rather, it may be entirely appropriate to *excuse* these farmers given their unfortunate circumstances (Francione, 2021).

Consider the following situation. Suppose I am a soldier who receives an order to airstrike a civilian target. Let’s suppose (quite simply) that these civilians are innocent, and that killing them will not help any just cause. Suppose that I refuse to do it, but my commanding officer puts a gun to my head and threatens to kill me unless I issue the order. In this situation, it would clearly be impermissible to airstrike the civilian target. But it seems appropriate to excuse me for issuing the airstrike given the duress I am under. That is, given the fact that someone is threatening to kill me unless I do some wrong action, it seems reasonable to excuse me for performing the action. I believe the same case could be made for farming animals in extreme poverty. Even if it is the case that killing animals is always impermissible, low-income farmers can be morally excused for doing so because of the duress they are under.

The above arguments show that taking option 2 of the trilemma is not as problematic as it might first appear. The anti-speciesist can make a plausible case for thinking that animals have stringent rights much in the same way that humans have stringent rights. If animals do have such stringent rights, then killing animals in cases of necessity would be impermissible. However, conceding this does not entail the implausible implication that farmers who kill animals out of necessity ought to be blamed or punished.^16^ Rather, it is very plausible that they should be morally excused, given the circumstances they happen to be in. These considerations lend strong credence to the idea that option 2 is a plausible way out of the trilemma. If we combine this conclusion with the conclusion that option 1 is also a plausible way out of the trilemma, then the trilemma dissolves into a merely apparent trilemma. Farming animals out of necessity is not as problematic for the anti-speciesist as it initially appeared.

## Conclusion

I have argued that a common justification for farming animals in extreme poverty, which appeals to the necessity of such a practice to meet the basic needs of those who engage in it, may be problematic for the anti-speciesist. I argued that such a problem forces the anti-speciesist into an apparent trilemma: either accept the permissibility of farming animals in cases of necessity while also conceding that it would be permissible to farm and kill humans in certain circumstances, reject the permissibility of farming animals in cases of necessity, or abandon the anti-speciesist position. I argued that the first and second options are both plausible ways out for the anti-speciesist, and so the trilemma dissolves.

It is important to note some limitations of this paper. Firstly, I have not definitively shown either option 1 or option 2 to be the correct option. If one wishes to defend option 1, one will need to make a stronger case than I have for thinking that it is permissible to kill humans in cases of necessity. If one wishes to defend option 2, one will need to make a stronger case than I have for thinking that animals have extremely stringent rights not to be killed. All that I have shown is that these options are plausible ways out of the apparent trilemma.

Furthermore, this paper does not give nearly enough recommendations for what ought to be done (if anything ought to be done) about farming animals in cases of necessity. Hundreds of millions of people farm animals in order to meet their basic needs. Deciding what ought to be done about this requires a multifaceted approach which appropriately takes into consideration the well-being of both farm animals (presuming they deserve moral consideration) and humans who need to farm animals in order to meet their basic needs. Some have suggested that small-holding farms (many of which containing livestock) will be key to rural development, hunger reduction, and poverty alleviation (HLPE, 2013; Samberg et al., 2016; Wiggins et al., 2010). But if animals deserve serious moral consideration, as anti-speciesists claim, then supporting small-scale farming (e.g. via trade) without ensuring improvements to animal wellbeing may be morally problematic. More work will need to be done to ensure that both animals and people who farm animals get the moral consideration they deserve.

Finally, it is important to emphasize that low-income animal agriculture is not a priority for anti-speciesists. If one cares about the suffering or killing of animals, then factory farming is by far the most significant target. It is estimated that 74% of all land livestock (23 billion animals at any given time) are factory farmed (Ritchie, 2023). Given the sheer scale of the issue, and given the horrible living conditions these animals face, it is hard to deny that this is one of the most significant moral challenges of our time. Furthermore, unlike farming animals in extreme poverty, it is unnecessary. We do not need factory farming to meet our nutritional, financial, or gustatory needs. For this reason, addressing factory farming (as opposed to low-income animal agriculture) is a far more tractable and desirable outcome for anti-speciesists.

## Data Availability

Not applicable.

## Footnotes

1 The World Bank defines extreme poverty as living on less than $2.15 per person per day at 2017 purchasing power parity (World Bank, 2022).

2 An estimation made in 2002 suggested that, out of over 1.33 billion people living in rural poverty, over 40% (almost 556 million) were livestock keepers (Thornton et al., 2002, pp. 53–68). However, the number of people living in extreme poverty today is closer to 600–700 million (Mahler et al., 2022). As such, the number of livestock keepers in extreme poverty will also be lower today.

3 This may also be a problem for those who are not anti-speciesists, but still think that killing animals for food is nearly always wrong. For the purposes of this paper, however, I will limit my discussion only to anti-speciesists.

4 A similar argument can be put forward to suggest that it is permissible for people to farm animals provided they *reasonably believe* it is necessary for meeting their basic needs. This position would entail that even if farming animals was not strictly speaking necessary for meeting one’s basic needs, one may be justified in farming animals provided they have evidence to suggest it is necessary. In this paper, I focus only on the objective version of this kind of argument – the argument that holds it is permissible to farm animals provided it is *objectively* (not merely “subjectively”) necessary for meeting one’s basic needs.

5 By “normal humans”, Singer is referring to humans which are not severely cognitively impaired.

6 Although I have yet to see any philosophers talk about *farming* humans out of necessity, some philosophers have discussed *killing* humans out of necessity, and the connection this has to anti-speciesist positions on subsistence hunting (Nobis, 2018).

7 I have Jean Kazez to thank for this point.

8 I have Dallas Havens to thank for this point.

9 Henry Salt makes this kind of response when rejecting this kind of argument being applied as a justification for humane animal agriculture (Salt, 1914).

10 This argument does not entail that it would be permissible to farm and kill humans or animals in non-necessity cases provided the lives of the farmed beings are good and the farmers benefit from the practice. Instead, the argument states two conditions for children farming to be justified: (1) the children have good lives, and (2) it prevents a significant amount of suffering for the farmer. Children farming in cases of necessity may meet both conditions, while children farming in non-necessity cases may not satisfy (2) if the benefits to the farmers are relatively trivial.

11 Note that even if we think it is impossible to say that existence is “better” for the children than non-existence, we can still conclude that a state of affairs in which they exist is better than one in which they don’t.

12 See Smolkin (2021) for a rejection of this kind of argument when applied to humane animal agriculture.

13 This is not a view I personally ascribe to, for reasons well defended in Sapontzis (2014). But since many philosophers seem to find it plausible, I think it is worth putting forward as a potential way for anti-speciesists to defend children farming out of necessity.

14 Killing animals in cases of necessity may be wrong even if animals lack such rights. For instance, if many animals need to be killed to save just one human, then it may be objectionable according to some consequentialist positions, e.g. a Prior-Existence Utilitarianism which maintains that outcomes should be evaluated depending on how they affect actual (instead of merely contingent) beings (Višak, 2013).

15 It should be noted that these philosophers often grant exemptions whereby these rights can be infringed in certain situations. For instance, some argue that it would be permissible to kill an innocent threat (e.g. a baby with a gun) in self-defence. They all agree, however, that it would be impermissible to kill an innocent *bystander* to save oneself. I take it that killing farm animals to meet one’s basic needs is more like killing an innocent bystander than killing an innocent threat.

16 It may, however, have the counterintuitive implication that farmers should be prevented from engaging in such a practice. Nevertheless, this is not a problem for our current state of affairs, where it would be absurd to prevent poor people from engaging in animal agriculture when rich people continue to exploit animals on a massive scale.
